# MMISH: Multicolor microRNA *in situ* hybridization for paraffin embedded samples

**DOI:** 10.1016/j.btre.2018.e00255

**Published:** 2018-05-01

**Authors:** Zhiyong Lei, Alain van Mil, Junjie Xiao, Corina H.G. Metz, Esther C.M. van Eeuwijk, Pieter A. Doevendans, Joost. P.G. Sluijter

**Affiliations:** a Department of Cardiology, Division Heart and Lungs, University Medical Center Utrecht, Utrecht, The Netherlands; b Regeneration and Ageing Lab, School of Life Science, Shanghai University, Shanghai, China; c Netherlands Heart Institute (ICIN), Utrecht, The Netherlands; d UMC Utrecht Regenerative Medicine Center, University Medical Center, Utrecht 3584CT, The Netherlands; e Central Military Hospital, Utrecht, The Netherlands

**Keywords:** TnI, Troponin I, LNA, locked nucleic acid, NBT/BCIP, combination of nitro-blue tetrazolium chloride and 5-bromo-4-chloro-3′-indolyphosphate *p*-toluidine salt, EDC, 1-Ethyl-3-[3-dimethylaminopropyl] carbodimide hydrochloride, MI, myocardial infarction, Tm, melting temperature, Colorimetric staining, Multicolor immunofluorescence staining, MicroRNA, *In situ* hybridization

## Abstract

To understand and assess the roles of miRNAs, visualization of the expression patterns of specific miRNAs is needed at the cellular level in a wide variety of different tissue types. Although miRNA *in situ* hybridization techniques have been greatly improved in recent years, they remain difficult to routinely perform due to the complexity of the procedure. In addition, as it is crucial to define which tissues or cells are expressing a particular miRNA in order to elucidate the biological function of the miRNA, incorporation of additional stainings for different cellular markers is necessary. Here, we describe a robust and flexible multicolor miRNA *in situ* hybridization (MMISH) technique for paraffin embedded sections. We show that the miRNA *in situ* protocol is sensitive and highly specific and can successfully be combined with both immunohistochemical and immunofluorescent stainings.

## Highlights

• A robust, sensitive and flexible multicolor miRNA *in situ* hybridization (MMISH) technique for paraffin embedded sections can be combined with both immunohistochemical and immunofluorescent staining.
• Usage of urea in our buffers which enhances the target-probe affinity by preventing intermolecular interaction within miRNAs or individual probes, and by reversing the EDC fixation induced epitope loss by denaturing the antigens, less toxic compared to toxic formamide.
• Second, it can be combined with immunofluorescent stainings, which allows one to analyze the expression and precise (sub)cellular location of the miRNA of interest.

## 1 Introduction

MicroRNAs (miRNAs) are short non-coding RNAs, which have been shown to play important roles in many different biological processes, pathological development and disease progression [1]. To explore the potential differential expression of one or many specific miRNAs in a physiological or pathological context, several techniques, such as Northern blot, qPCR, microarray, and next generation deep sequencing technologies can be used [2]. However, these techniques will provide the levels using a mixed sample of different cell types. In addition, not only expression levels, but also their locations, the cell type identification within the tissue are important [3]. A miRNA can only fulfill its function when its expression is temporal-spatial correlated with its targeted mRNAs. Thus a robust technique to define the expression patterns of specific miRNAs at the cellular level is crucial to elucidate their functions [[4], [5], [6], [7], [8], [9]].

One way to visualize miRNAs at the cellular level is by performing miRNA *in situ* hybridization [10,11]. The concept is straightforward and in general similar to the traditional *in situ* hybridizations for long coding mRNAs: a pre-labeled nucleic acid sequence, called probe, complementary to the selected miRNA is used to visualize the localization of the specific miRNA. Based on these well-established mRNA *in situ* procedures, several protocols have been developed to detect miRNA expression, which have advanced our understanding of how and where miRNAs are located [[12], [13], [14], [15], [16], [17], [18]].

MiRNA *in situ* techniques have been greatly improved and made less complicated, but even so, they are still laborious and difficult to perform routinely. Several modifications have been implemented to improve the sensitivity of the technique: 1) the use of Locked Nucleic Acid (LNA) based probes has significantly improved the hybridization signal and reduced the background [13]; 2) by using double-labeled probes, increased signal-to-noise ratios have been achieved; 3) by the introduction of an extra 1-Ethyl-3-[3-dimethylaminopropyl] carbodiimide hydrochloride (EDC)-based fixation step, free miRNAs were prevented from escaping into hybridization buffers [18]. However, one major drawback of EDC fixation is that it destroys the epitope of cell surface markers, which makes it difficult to perform subsequent immunohistochemical staining [18]. Still, to date only highly abundant miRNAs have been localized and defined by *in situ* hybridization [[12], [13], [14], [15], [16], [17], [18]], suggesting still low sensitivity of this technique and the need for further optimization. Moreover, most of the available protocols require cryopreserved tissue samples [12,13], while most clinical grade patient samples are paraffin embedded to better preserve morphology.

**Fig. 1:**
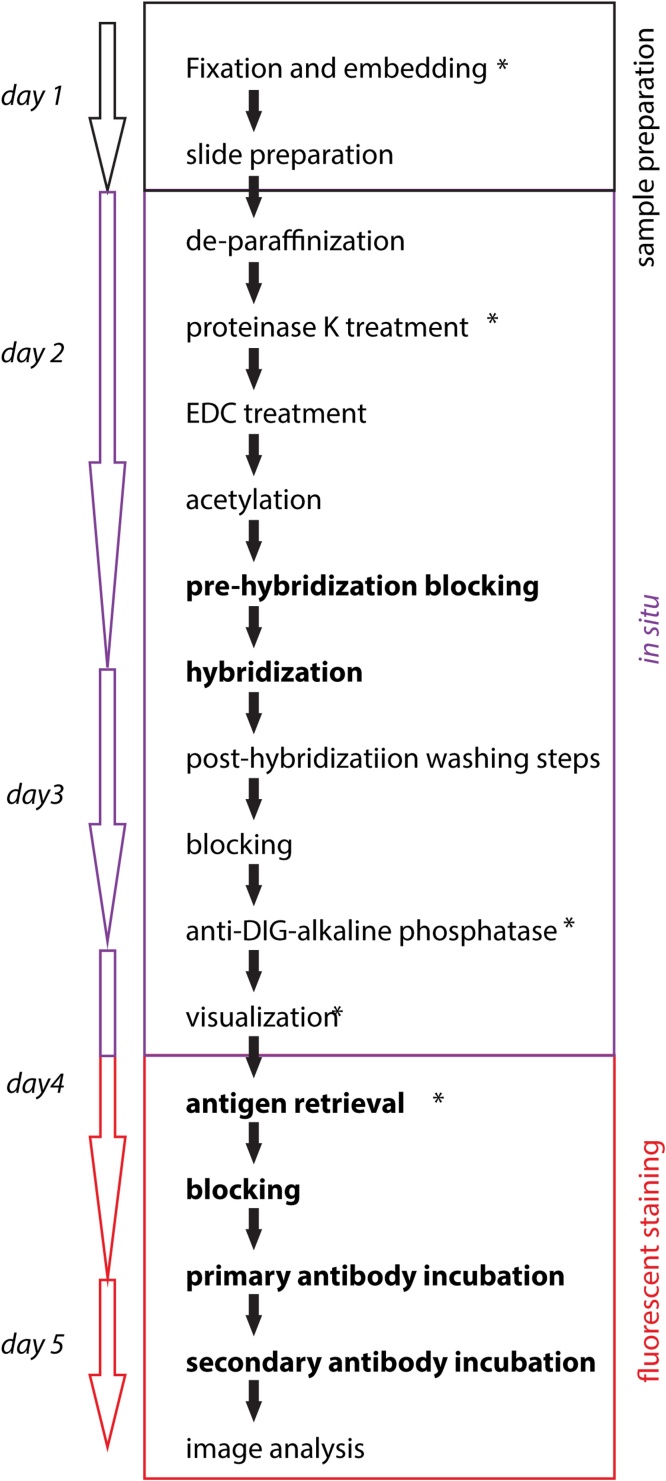
The workflow of MMISH which covers most of the critical points presented in this paper, including the time needed for each steps and sections. Steps marked with a * require optimization. Steps with bold characterization contain differences from other reported methods.

Here, we provide a non-toxic urea-based miRNA *in situ* hybridization protocol for paraffin embedded samples in combination with different visualization methods. By using our protocol, a multicolor image can be created by combining high sensitive *in situ* hybridization with immunofluorescent stainings, thereby allowing to visualize the expression of miRNAs at the cellular and even subcellular level (Fig. 1).

## 2 Results and discussion

**Fig. 2:**
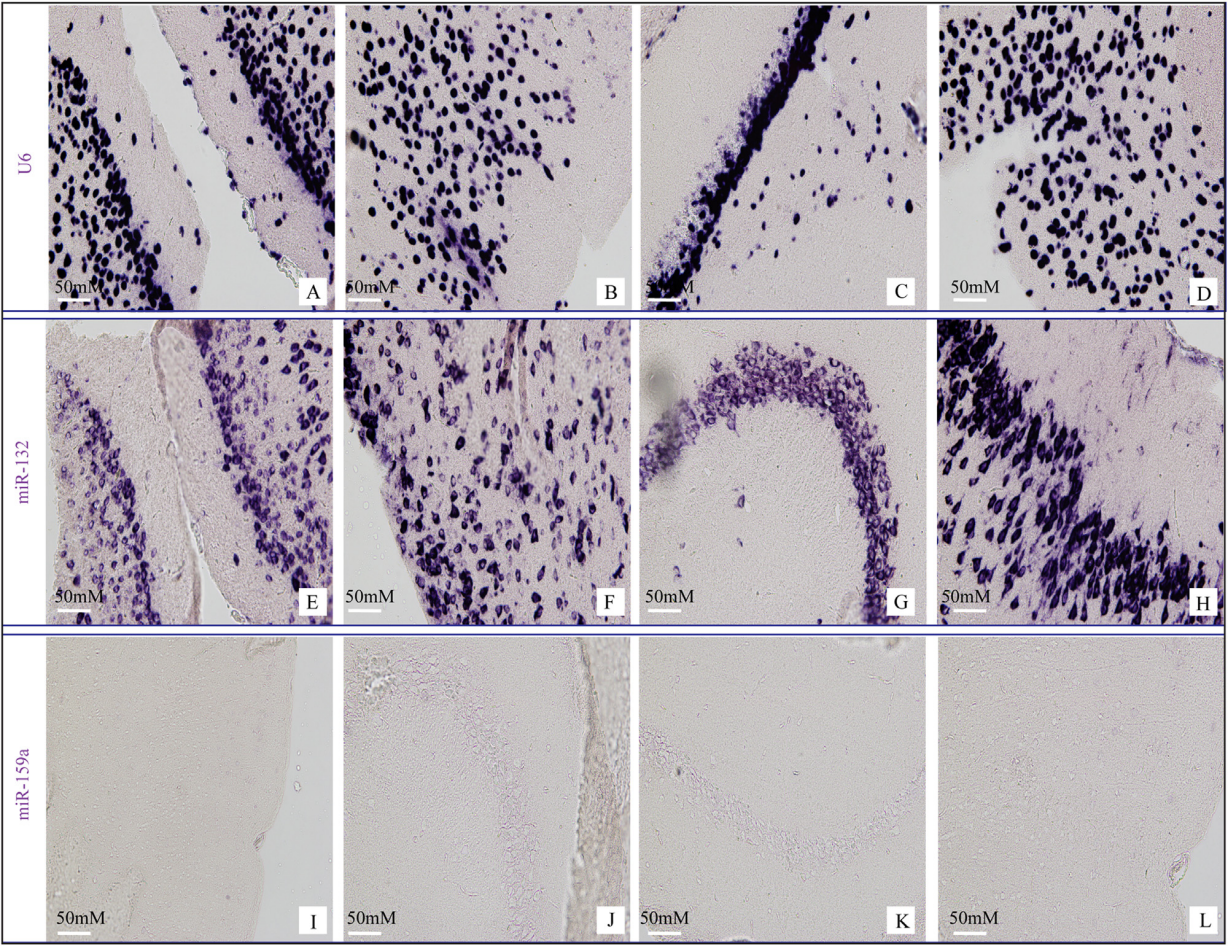
Representative images of miRNA *in situ* hybridization for U6 (A, B, C and D), miR-132 (E, F, G and H) and miR-159a (I, J K and L) in paraffin embedded mouse brain. U6 is used as a positive control and plant specific miR-159a serves as a negative control.

**Fig. 3:**
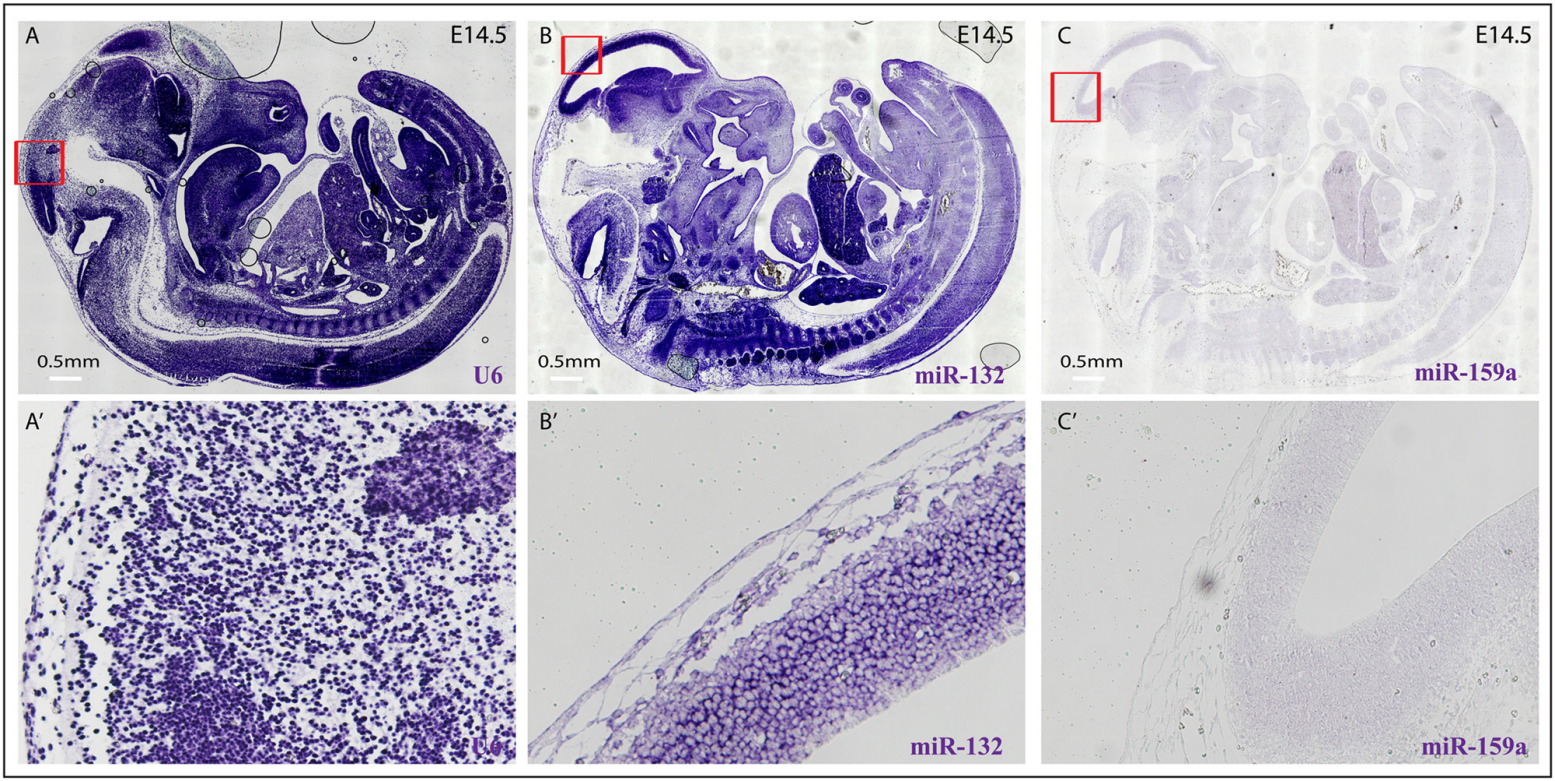
Representative images of miRNA *in situ* hybridization for U6 (A), miR-132 (B) and miR-159a (C) in paraffin embedded mouse embryo at E14.5. U6 is used as a positive control and plant specific miR-159a serves as a negative control. Figure A', B' and C' show high magnifications of the red box indicated areas in A, B and C.

To show the specificity and feasibility of our *in situ* protocol, we first performed miR-132 *in situ* hybridization on paraffin embedded mouse brain [11,[22], [23], [24]]. As expected, miR-132 is expressed in the cytoplasm of neural cells, compared to the exclusive nuclear location of U6 (Fig. 2). miR-159a, a plant specific miRNA, which is not present in mammalian cells, is used here as a negative control. In addition, we performed *in situ* hybridization for U6, miR-132 and miR-159a on paraffin embedded mouse embryo (E14.5) sections. As expected, U6 showed a strong nuclear staining throughout the embryo; miR-132 showed high expression in the brain, but is visible in other tissues as well (Fig. 3)[[22], [23], [24], [25], [26], [27], [28], [29], [30]].

**Fig. 4:**
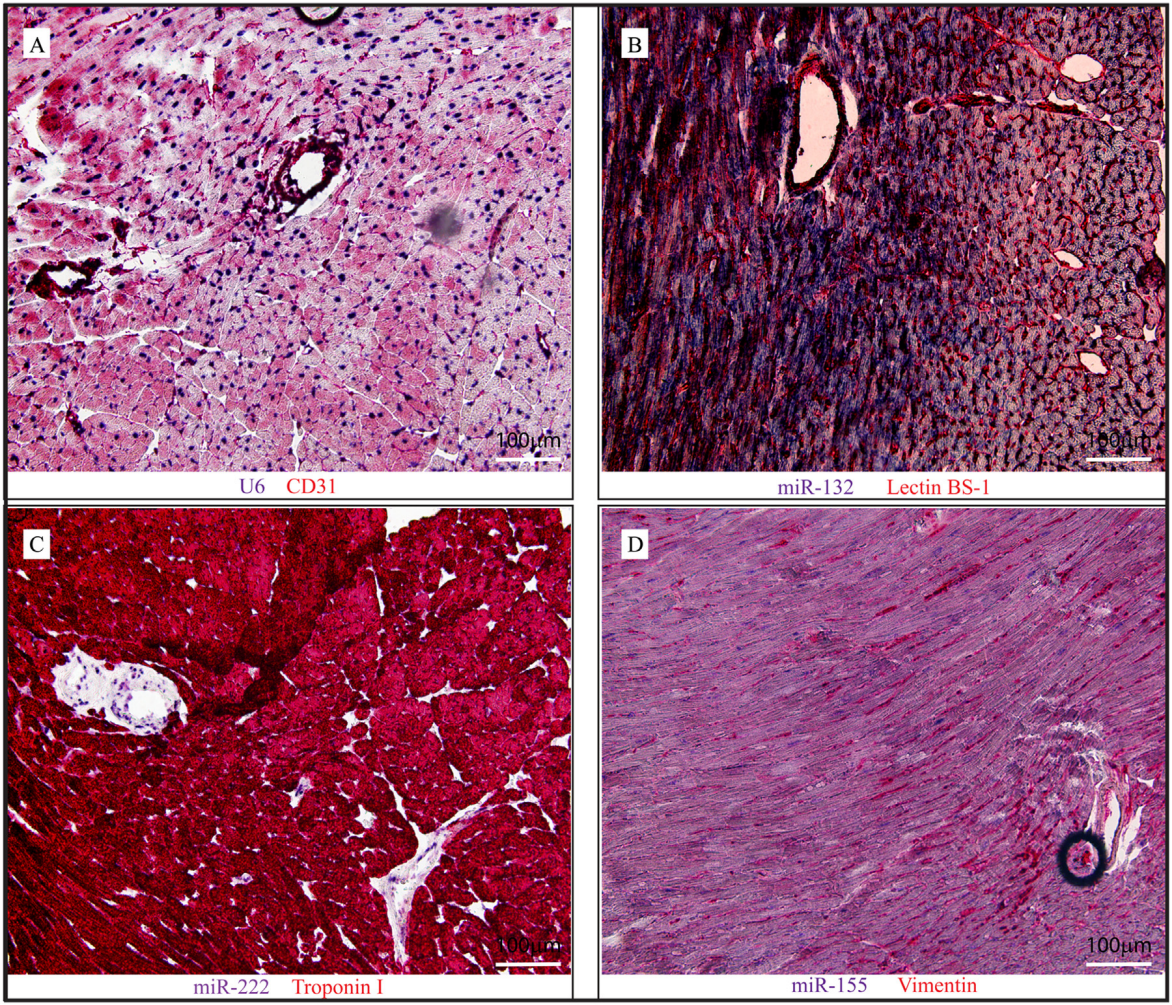
Representative images of miRNA *in situ* hybridization for U6 (A), miR-132 (B), miR-222 (C) and miR-155(D) in paraffin embedded mouse heart in combination with immunohistochemical stainings. A: endothelial cells (CD31), B: endothelial cells (lectin BS-1), C: cardiomyocytes (Troponin I) and D: (myo)fibroblasts (Vimentin). (miRNAs in purple/blue, cell-specific markers in red).

**Fig. 5:**
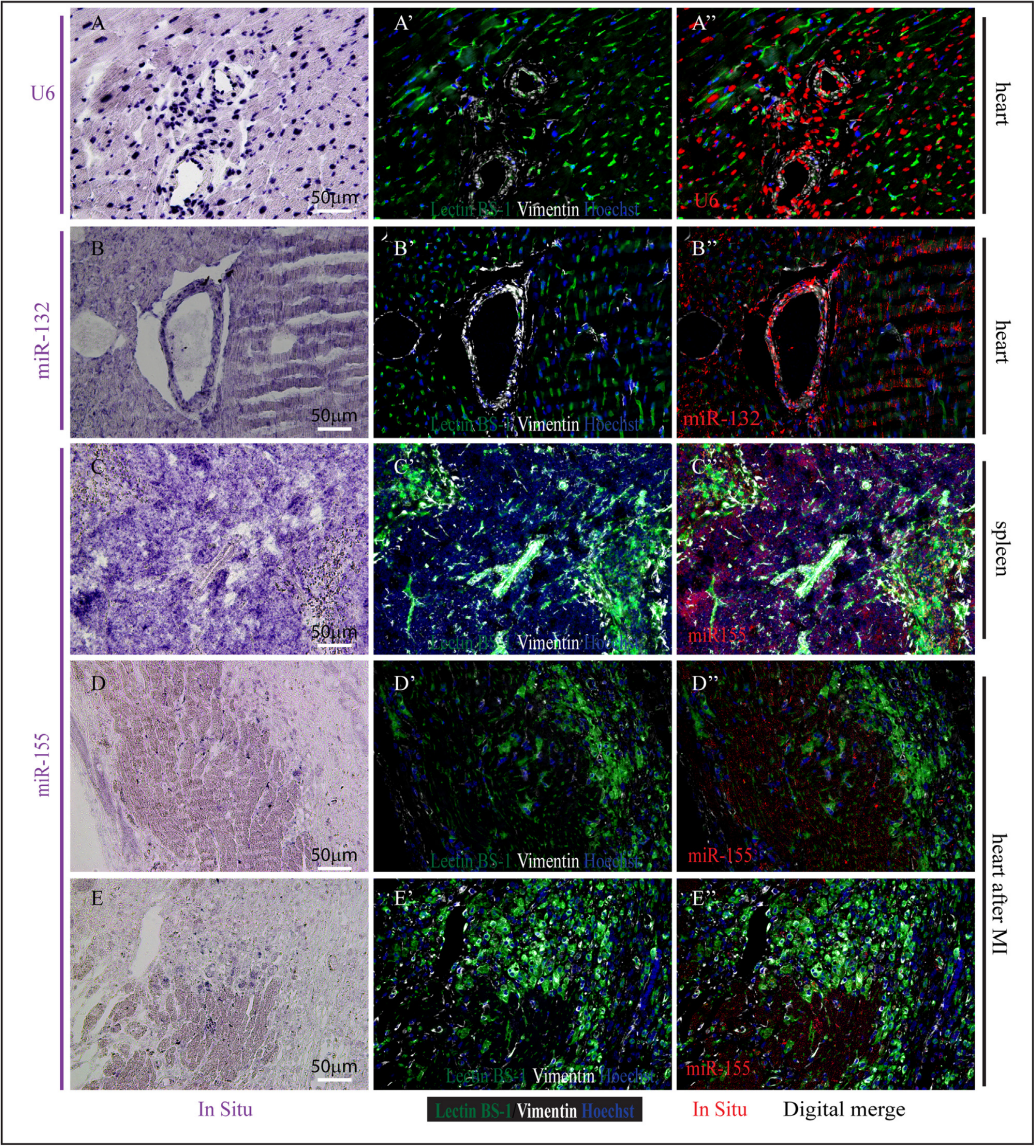
Representative images of miRNA *in situ* hybridization for U6 (A), miR-132 (B) and miR-155 (C, D and E) in paraffin embedded healthy mouse heart (A, B), spleen (C) or myocardial infarcted heart (D, E) in combination with immunofluoresent stainings. Nuclei are stained with Hoechst, endothelial cells with Lectin BS-1 in green, and (myo)fibroblasts in white. Digital merged images from *in situ* and immunofluorescent signals are shown on the right with nuclei in blue; endothelial cells in green; (myo)fibroblasts in white, and the *in situ* signal digitally converted into red.

Subsequently, we showed that our *in situ* protocol can be combined with additional colorimetric stainings. Immediately after the miRNA *in situ* section, antigen retrieval was performed with "antigen retriever" as described previously [31]. The high temperature stops alkaline phosphatase activity of the miRNA probes and helps the cellular surface epitope to recover. After *in situ* hybridization for U6, miR-132, miR-222 and miR-155 in cardiac tissue, we identified the cellular types by immunofluorescent staining for CD31 (Endolthelium), Lectin BS-1 (Endothelium), cardiac Troponin I (Cardiomyoyctes) and Vimentin (Fibroblasts), respectively. As shown in Fig. 4, the *in situ* hybridization miRNA signal is shown in purple/blue, whereas the cell types by antibody staining were visualized *via* chromogen in red. We show that miR-132 in expressed predominantly in cardiomyocytes, miR-222 is present in the nuclei of the smooth muscle cells, while miR-155 is lowly expressed in cardiomycoytes. Depending on the combination, signals are sometimes difficult to distinguish from one another. We therefore set up a protocol where we can combine the colorimetric *in situ* hybridization with immunofluorescent stainings for cell-specific markers (Fig. 5).

As described above, we first performed *in situ* hybridization for U6, miR-132 and miR-155 on various paraffin embedded mouse tissues, including heart and spleen, and subsequently stained these sections to visualize endothelial cells (Lectin BS-1) and myofibroblasts (Vimentin). The signals from the different channels were digitally merged into a 4-channel fluorescent image by using ImageJ. U6 is exclusively localized in the nuclei, miR-132 is expressed in the cardiomyocytes and large vessels (Vimentin positive), and miR-155 is highly expressed in B cells in the spleen which are small, lectinBS-1 and Vimentin negative cells, in some small cells in the infarcted area and is also detectable in cardiomyocytes after myocardial infarction.

As in other *in situ* hybridization protocols, there are many crucial steps and some steps must be optimized case by case. The time needed for PFA fixation before embedding can be different depending on the type of tissue and the size of the tissue. Fixation duration will also influence the time needed for proteinase K treatment and antigen retrieval later on. Therefore, the concentration and time of proteinase K treatment should be optimized. In practice, after proteinase K treatment, a quick Hoechst staining is very helpful as, in general, a bright nuclear signal with a clear nuclear edge suggests proteinase K treatment is optimal. Additionally, the hybridization temperature should be determined experimentally. The annealing temperature provided by the supplier is determined *in silico* and can be significantly different in reality. At last, the time for development of the *in situ* signal differs between different miRNAs and tissues, as it is directly related to the abundance of the targets.

### 2.1 Notes

We used urea in our buffers which has previously been used successfully in RNA gel electrophoresis to prevent RNA from forming secondary structures [33], and recently, also for antigen retrieval [34]. Thus, urea may play a dual role by keeping the miRNA molecules and probes linear, thereby enhancing the target-probe affinity by preventing intermolecular interaction within miRNAs or individual probes, and by reversing the EDC fixation induced epitope loss by denaturing the antigens.

A great advantage of our protocol is *in situ* hybridization can be combined with immunofluorescent stainings. In the final steps of the *in situ*, NBT/BCIP is converted into a dark blue stable crystal by alkaline phosphatase. This signal is so stable that it is still present after autoclave treatment. This makes it possible to follow-up with standard immunofluorescent stainings, and allows different antigen retrieval procedures. Our protocol is not limited to paraffin embedded samples only and will also work for cryopreserved samples. In our experience, cryosections usually show stronger signals than paraffin-embedded tissues. However, a well-performed cryopreservation procedure is critical to prevent RNA degradation and cryo-damaging of the tissue during freezing.

### 2.2 Limitations

We found that nuclear Hoechst signals can be affected when NBT/BCIP was used for detection of U6 in the *in situ* hybridization due to steric hindrance of abundant overlaying signals. Moreover, this technique is semi-quantitative: one can compare the expression of a specific miRNA between different conditions, but direct comparison of two different miRNAs is limited unless the labeling property of different probes have been proven to be the same.

### 2.3 Application and advantages over other methods

Our protocol uses urea in the hybridization buffer, as compared to toxic deionized formamide, and it can be easily combined with different immune-labeling procedures, which allows one to analyze the expression and precise (sub)cellular location of the miRNA of interest.

## Ethical standards

The use of mice tissue was granted by the Animal Ethical Experimental committee of Utrecht University and was performed under the Guide for the Care and Use of Laboratory Animals.

## Disclosures

The authors have declared that no competing interests exist.
