# Supplementary material for: MMISH: Multicolor microRNA in situ hybridization for paraffin embedded samples
Source: Biotechnol Rep (Amst). 2018 May 1;18:e00255. doi: 10.1016/j.btre.2018.e00255 (PMC5989586; doi:10.1016/j.btre.2018.e00255)
Supplement: Supplementary file 1 [file mmc1.docx]

**Supplementary**

**A step by step guide:**

**Materials and Methods:**

REAGENTS

Tissue-Tek® Tissue-Clear® Xylene Substitute (Cat. 1426, SAKURA), proteinase K (Cat. 03115828001, Roche); PBS 1x (Cat. [10010023](http://www.lifetechnologies.com/order/catalog/product/10010023), Life Technologies); Tris (Cat. T1503); glycine (Cat. G8898, Sigma); Paraformaldehyde (PFA) (Cat. P-6148, Sigma), Diethylpyrocarbonate (DEPC) (Fluka, 32490); Triethanolamine (Fluka, 90279); HCl (Riedel, 30721); Acetic anhydride (Sigma, A6404); 50x Denhardt’s (Cat. 750018, Life technologies); Yeast tRNA (Cat. [10109495001](http://lifescience.roche.com/shop/en/us/products/trna-362321-1), Roche) Anti-digoxigenin antibody (Roche, 1093274); Levamisole (Cat. X3021, DAKO); NBT/BCIP (Cat. K0598, DAKO); chromogen (Cat. K0640, DAKO); Fast Red Substrate (Dako, K0699); CHAPS (Sigma, C5070-5G); Tween (Cat. 93773, Fluka); NaCl (sigma); MgCl2 (Cat. 2320946, sigma); 20 X SSC (Cat. 15557-044, Life technologies); fluoromount-G (Cat.0100-1, SourthernBiotech); 1-Ethyl-3-[3-dimethylaminopropyl]carbodiimide hydrochloride (EDC) (Cat. 77149, Thermo Fisher); Heparin (Cat. H4784, Sigma)

.

*Used antibodies*: Anti-Digoxigenin-AP, Fab fragments antibody (Cat. 11093274910 Roche); anti-CD31 (Cat. 550274, BD Pharmingen); anti-cardiac Troponin I(Cat. ab47003, Abcam); anti-Vimentin (Cat. ab45939, Abcam); anti-Actinin (sarcomeric) (Cat. A7811, Sigma); Hoechst (Cat. H1399, Life technologies); Lectin BS-1 (Cat. L2895, Sigma).

*5'- and 3'- double DIG-labeled miRCURY LNA™ Detection probes*: miR-132 (Cat. 38031-15, Exiqon), miR-222 (Cat. 38499-15, Exiqon), miR-155 (Cat. 88083-15, Exiqon), miR-25 (Cat. 18122-15, Exiqon), miR-159a (Cat. 99003-15, Exiqon), and U6 (Cat. 99002-15, Exiqon).

EQUIPMENT
Coverslips (Menzel-Gläser, 24 x 60 mm)
Antigen retriever 2100 (Aptum Biologics Ltd)
Microscope (Olympus, BX53)

REAGENT SETUP

1. DEPC-treated PBS (1 liters): Add 1 ml of DEPC to 1 liters of PBS and mix vigorously. Keep at 37ºC overnight, then autoclave.
2. DEPC-treated water (1 liter): Add 1 ml of DEPC to 1 liter of milliQ water, keep at 37ºC overnight, then autoclave.
3. Proteinase K digestion buffer: 5 mM Tris, 1 mM EDTA, 1 mM NaCl, pH 7.4
4. EDC fixation buffer: 0.13 M 1-methylimidazole in 0.3 M NaCl, pH 8.0.
5. Acetylation buffer: Add 6.67 ml triethanolamine, 875 μl HCL (37%), and 1.25 ml acetic anhydride in 493 ml H_2_O.
6. Pre-hybridization blocking buffer: Urea (2 M), 2.5x SSC, 1x Denhardt’s, 200 µg/ml yeast tRNA, 0.1% CHAPS, 0.1% Tween, 50 ug/ml heparin.
7. *In situ* hybridization buffer: Urea (2 M), 2.5x SSC, 1x Denhardt’s, 200 µg/ml yeast tRNA, 0.1% CHAPS, 0.1% Tween, 50 ug/ml heparin.
8. Blocking buffer: 0.5% BSA, 10% normal goat serum in TBST (0.1% tween 20).
9. Antigen retrieving buffer: 10 mM Tris Base, 1 mM EDTA Solution, 0.05% Tween 20, pH 9.0.
10. Primary and secondary antibody incubation buffer: 0.5% BSA, 10% normal goat serum in TBST (0.1% tween 20).
11. Washing solution I: 0.1 M Tris, pH 7.5, 0.15 M NaCl, 0.1% Tween 20.
12. Washing solution II: 0.1 M Tris, pH 9.0, 0.15 M NaCl.

Slide preparation

1. Fix adult tissue in freshly prepared 4% PFA at 4ºC overnight under rotation. The fixation time for embryonic tissue can be reduced to 1 hour.
2. Cut paraffin embedded samples at 10 µm, dry the section for 1 hour at a 56 ºC plate.
3. Start the deparaffinization with fresh tissue clear for 2x 10 min.
4. Rehydrate sections subsequently with 99% ethanol, 95% ethanol, 70% ethanol, 50% ethanol, and PBS. 5 min for each step.

Proteinase K treatment

1. Optional: Delimit the sections with a DAKO Pen.
2. Transfer the slides in their holder into pre-warmed Proteinase K solution (5 mg/ml, 37°C) for 7 min.
3. Wash slides with 0.2% glycine in PBS for 2x 10 min to remove Proteinase K.

EDC fixation

1. Wash slides in EDC fixation buffer for 2x 10 min to remove residual phosphate groups from PBS.
2. Cover section with 200 ul 0.16 M EDC solution in EDC fixation buffer for 1 hour at room temperature.
3. Wash slides for 10 min to remove residual EDC with 0.2% glycine in PBS.

Acetylation

1. Transfer to acetylation solution for 30 min at room temperature.
2. Wash slides in washing solution I for 10 min.

Hybridization

1. Apply 200 μl pre-hybridization buffer on each slide at room temperature for 1 hour.
2. Recover probe in appropriate amount of hybridization buffer. The final concentration is probe specific, depending on the abundance of the miRNA of interest and the affinity of the probe to the target. For U6 5 nM is sufficient, miRNA probes were used at 10 nM. We recommend to first test a range of 1 to 40 nM.
3. Replace the pre-hybridization buffer with 100 ul hybridization solution, including the probe and cover the section with a 60 mm x 24 mm coverslip to avoid evaporation.
4. Place slides on a 65ºC hot plate to denature the probe and miRNA targets.
5. Incubate slides overnight at 30ºC below their Tm, as provided by the supplier. For example, Tm (miR-132) is 86ºC, Tm (miR-155) is 76ºC, Tm (miR-222) is 88ºC, and Tm (miR-159) is 87 º C. Therefore, the sections for miR-132 are incubated at 56ºC, miR-155 at 46ºC, miR-222 at 58ºC and miR-159a at 57 ºC.
6. Soak slides in pre-warmed 5x SSC and carefully remove coverslips at their hybridization temperature.
7. Wash slides 2 times in 5x SSC, 10 min each at their hybridization temperature.
8. Wash slides for 4 times in 0.2x SSC, 10 min each at their hybridization temperature.
9. Wash slides in wash buffer I for 10 min at room temperature.

Staining

1. Block sections by applying 0.5% BSA and 10% normal goat serum in TBST for 1 hour.
2. Remove the blocking solution and apply 100 ul anti-DIG-alkaline phosphatase antibody in 5% BSA (1:1500) for 1 hour at room temperature.
3. Wash slides for 2 times in washing solution I, 5 min each.

Visualization

1. Wash slides for 10 min in washing solution II. Mix 3 ml BCIP/NBT solution with 3 drops levamisole solution, apply 200 μl to each slide and incubate at room temperature.
2. Regularly check the staining under the microscope. The incubation time needed for each probe will differ, depending on the expression level of each miRNA/ probe sensitivity. For high abundant targets, such as U6, a strong nuclear staining is visible within 15 min, but for low abundant miRNAs the incubation time can be extended to several hours. However, incubation time longer than 6 hours usually hardly improves the signal further.

Immunofluorescent staining

1. Wash slides in TBS for 5 min and place the slides in an antigen retrieval chamber, filled with appropriate antigen retrieval buffer, place the chamber holder in antigen retriever 2100, turn on the antigen retriever. It takes about 2 hours for the antigen retriever to complete the procedure and cool down.
2. Take out the slides and wash them in TBS for 10 min.
3. Optional: delimit the sections again with a DAKO pen.
4. Block with 0.5% BSA and 10% normal goat serum in TBST for 1 hour at room temperature.
5. Dilute antibodies in blocking solution, apply 100 ul to the slides and incubate overnight at 4ºC .
6. Wash slides 3 times in washing buffer I, 10 min each, with gentle agitation.
7. Dilute appropriate fluorescent-labeled secondary antibodies and apply to the sections for 1 hour at room temperature.
8. Wash slides 3 times in washing buffer, 10 min each, with gentle agitation.
9. Dilute Hoechst (1 mg/ml) in PBS and apply to the slides for 10 min at room temperature.
10. Wash slides 2 times in washing buffer I, 5 min each.
11. Apply 100 ul fluoromount-G solution and seal slides with 24 mm x 60 mm coverslips.
12. Dry the slides in the dark. When dry, they are ready for microscopy.
